# Supplementary material for: High Prevalence and Persistence of Escherichia coli Strains Producing Shiga Toxin Subtype 2k in Goat Herds
Source: Microbiol Spectr. 2022 Aug 8;10(4):e01571-22. doi: 10.1128/spectrum.01571-22 (PMC9431244; doi:10.1128/spectrum.01571-22)
Supplement: Supplemental file 2 — Supplemental material. Download spectrum.01571-22-s0002.pdf, PDF file, 2.8 MB [file spectrum.01571-22-s0002.pdf]

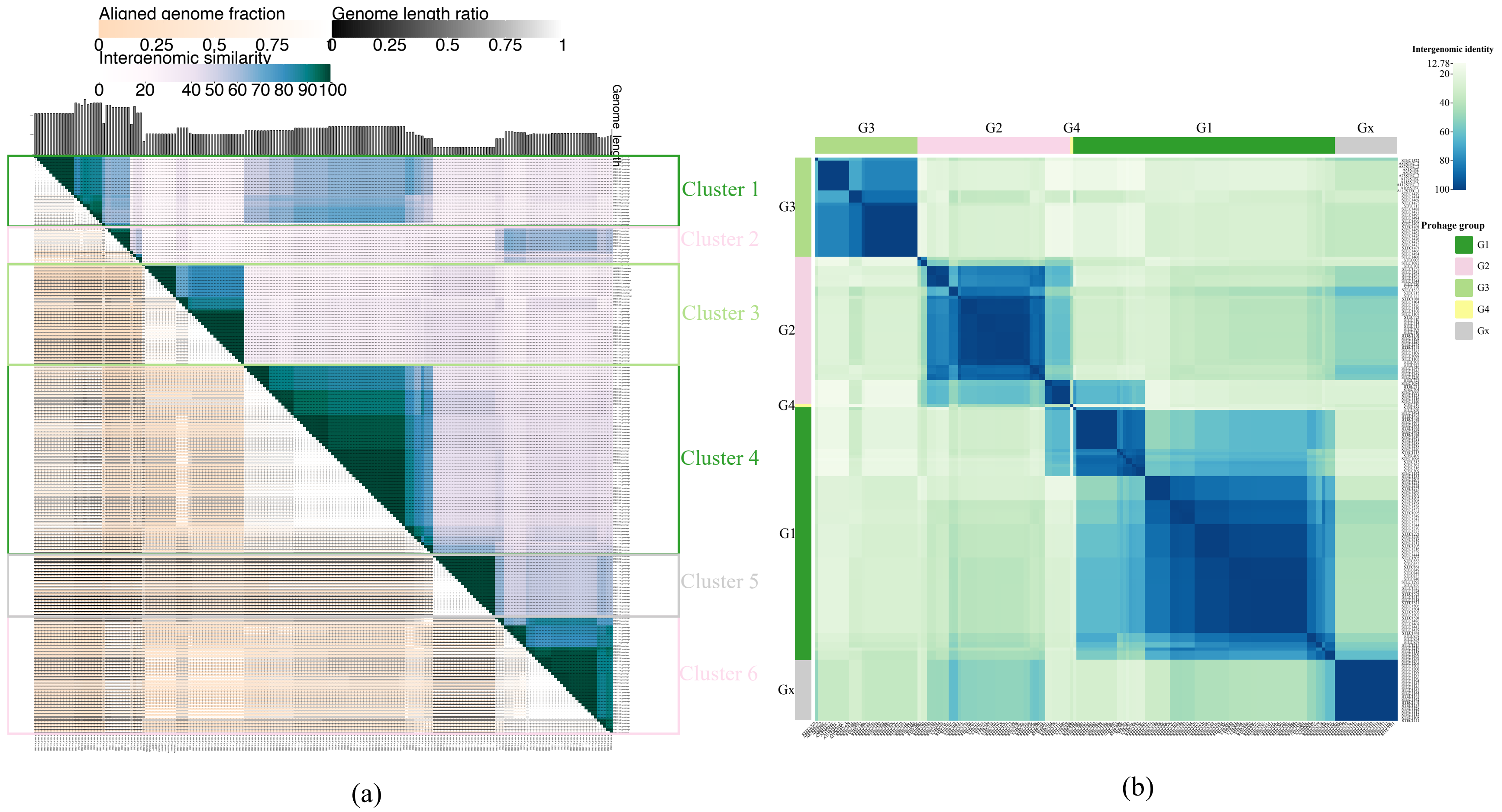

Figure S1. (a). VIRIDIC heatmap incorporating intergenomic similarity values (right half) and alignment indicators (left half and top annotation) of Stx2k-converting prophages. Colored rectangle indicates hierarchical clusters. (b). Heatmap of pairwise intergenomic distances of 187 Stx2k-converting prophages assigned as different phage groups. The comparison, clustering and intergenomic similarity values between pairs of prophage genomes were performed using VIRIDIC. Online tool ChiPlot (https://www.chiplot.online/#Phylogenetic-Tree) was used to visualize and annotate the heatmap. The colored bar represents Stx2k prophage groups.

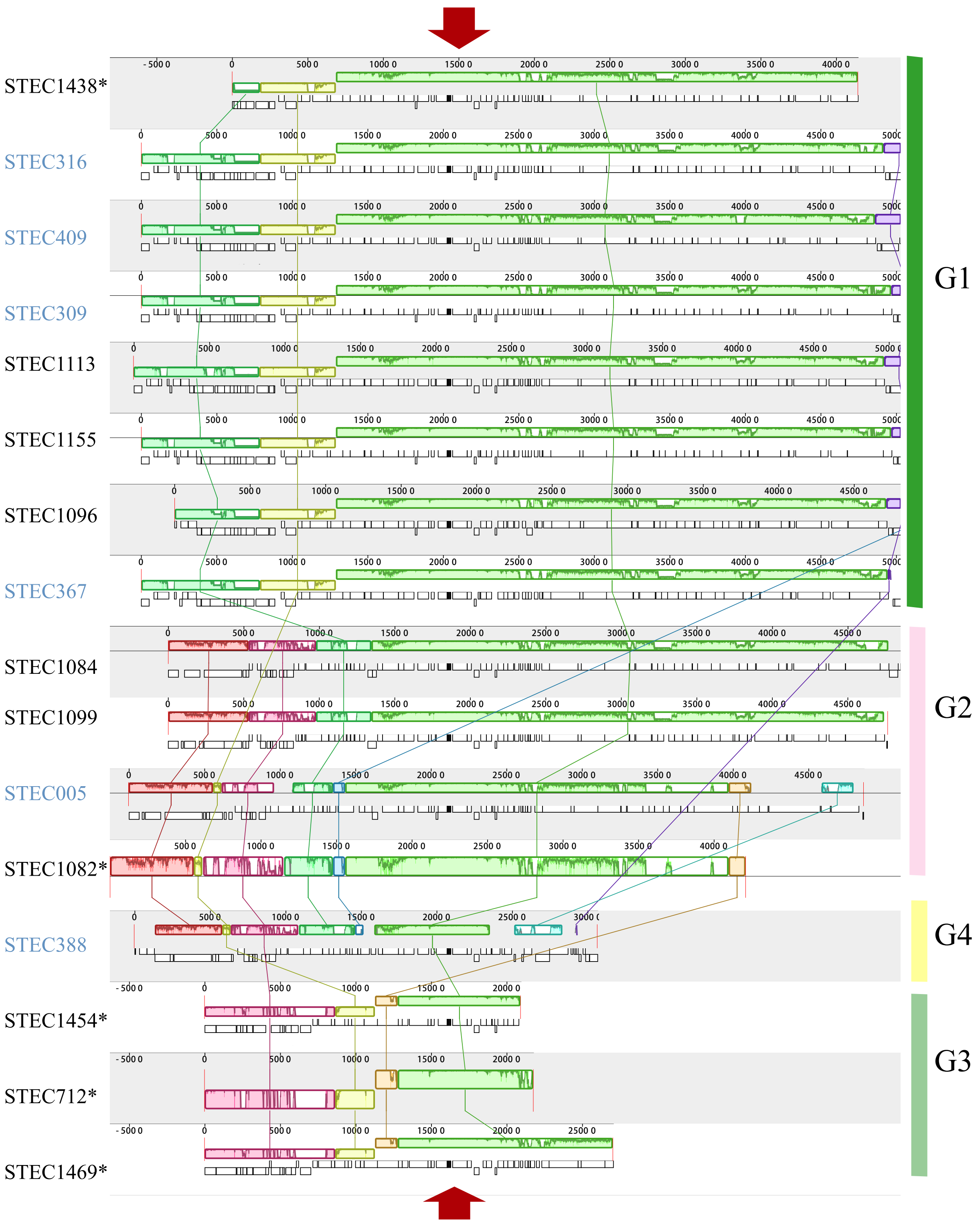

Figure S2. Sequence comparison of the Stx2k-converting prophages from Stx2k-STEC strains representative of different serotypes and sequence types. Phage sequences extracted from the genomes of the Stx2k-STEC isolates were subjected to sequence analysis using Mauve. Similar color denotes regions of shared sequence, and the height of the bars denotes level of similarity of the shared sequence regions. The color of the text indicates the source of strains, black represents goat-derived strains in this study, and light blue represents other source-derived strains in other studies. The asterisk (*) signifies incomplete prophages. The location of the *stx* genes is shown with a red arrow. The colored bar in the right represents the four prophage groups.

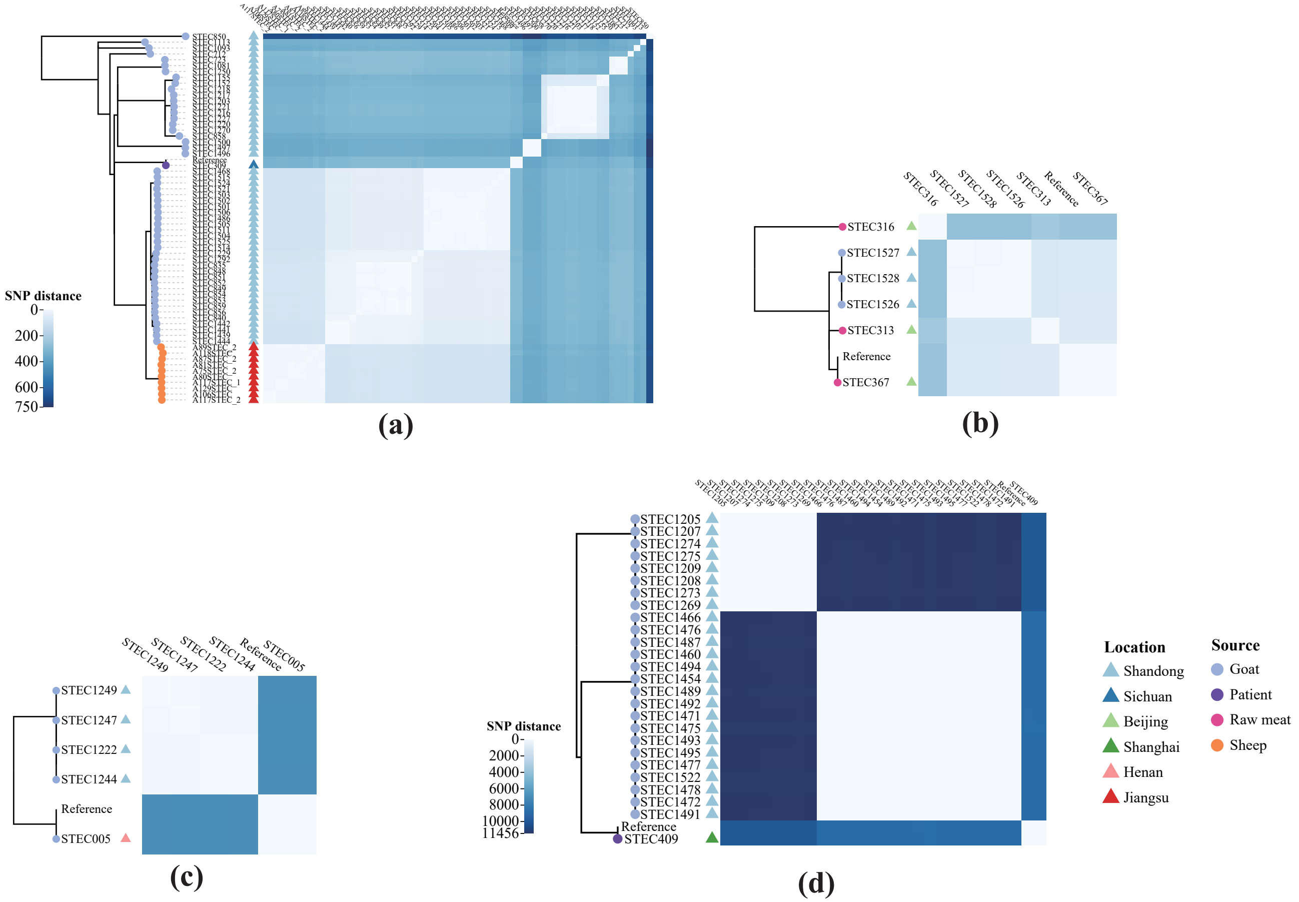

Figure S3. SNP differences of Stx2k-STEC strains within same phylogenetic clusters. Heatmap illustrating pairwise SNP distances for (a) 62 isolates and reference strain STEC309 (NZ_CP041435.1) in cluster 1, (b) 6 isolates and reference strain STEC367 (NZ_CP041429.1) in cluster 3, (c) 5 goat-derived isolates from different regions and reference strain STEC005 (NZ_CP041437.1) in cluster 4, (d) 26 isolates and reference strain STEC409 (NZ_CP041422.1) in cluster 5. The maximum likelihood trees presented as cladograms, constructed using all isolates and reference strain in each cluster as mentioned above. All trees were constructed using SNP alignments.
